# The changing epidemiology of trauma in child-bearing age women

**DOI:** 10.1186/s13017-023-00495-7

**Published:** 2023-03-29

**Authors:** Fikri M. Abu-Zidan, Hani O. Eid, David O. Alao, Hassan Elbiss

**Affiliations:** 1 The Research Office, College of Medicine and Health Sciences, United Arab Emirates University, Al-Ain, United Arab Emirates; 2 Rescue and Air Ambulance, Abu Dhabi Police Aviation, Abu Dhabi, United Arab Emirates; 3 Department of Internal Medicine, College of Medicine and Health Sciences, United Arab Emirates University, Al-Ain, United Arab Emirates; 4 Emergency Department,, Tawam Hospital, Al-Ain, United Arab Emirates; 5 The Department of Obstetrics and Gynecology, College of Medicine and Health Sciences, United Arab Emirates University, Al-Ain, United Arab Emirates

**Keywords:** Injury, Trauma, Prevention, Mechanism, Women, Child-bearing age, Pregnancy, Incidence

## Abstract

**Background:**

In the last two decades, there have been major improvements in the trauma system in the United Arab Emirates (UAE). We aimed to study the changes in the incidence, type, severity, and outcome of trauma of hospitalized child-bearing age women in Al-Ain City, UAE, during that time.

**Methods:**

Data from two separate trauma registries of Al-Ain Hospital, which were prospectively collected from March 2003 to March 2006 and January 2014 to December 2017, were analyzed retrospectively. All women aged 15–49 years were studied. The two periods were compared.

**Results:**

Trauma incidence of hospitalized child-bearing age women was reduced by 47% during the second period. There were no significant differences in the mechanism of injury between the two periods. Road traffic collision was the main cause of injury (44% and 42%, respectively) followed by fall down (26.1% and 30.8%, respectively). The location of injury was significantly different (p = 0.018), with a strong trend of more home injuries in the second period (52.8% compared with 44%, p = 0.06). There was a strong statistical trend of mild traumatic brain injury (GCS 13–15) in the second period (p = 0.067, Fisher’s Exact test). Those who had normal GCS of 15 were significantly higher in the second period compared with those in the first period (95.3% compared with 86.4%, p < 0.001, Fisher’s Exact test) despite having more anatomical injury severity of the head (AIS 2 (1–5) compared with 1 (1–5), p = 0.025). The NISS was significantly higher in the second period (median (range) NISS 5 (1–45) compared with 4 (1–75), p = 0.02). Despite that, mortality was the same (1.6% compared with 1.7%, p = 0.99) while the length of hospital stay was significantly less (mean (SD) 5.6 (6.3) days compared with 10.6 (13.6) days, p < 0.0001).

**Conclusions:**

The incidence of trauma in hospitalized child-bearing-age women was reduced by 47% over the last 15 years. Road traffic collisions and falls are the leading cause of injury in our setting. Home injuries increased over time. The mortality remained stable despite the increased severity of injured patients. More injury prevention efforts should target home injuries.

## Introduction

Trauma causes 8% of deaths worldwide, which mainly affects young adults [1]. Around 70% of the world's population gets injured at least once [2], and one-third are women. [3]. Nearly half of the women in developed countries drive cars [4]. The most common non-obstetric trauma encountered by pregnant women is caused by road traffic collisions (RTCs), leading to maternal and fetal deaths [5, 6]. A recent systematic review showed that studies on maternal trauma due to motor vehicle crashes are retrospective, having high risk of selection bias [7].

The United Arab Emirates (UAE) is a high-income developing country with rapid major construction projects, increased number of road highways, and increased road vehicles [8]. Women in the UAE constitute 70% of the university graduates, run two-thirds of the public sector jobs, and fill 30% of its leadership positions [9]. This increases their outdoor activities, including car driving. Injury is the second cause of death in the United Arab Emirates (UAE) [10]. A trauma system is a pre-planned and organized effort to reduce the trauma death, disability, and its direct and indirect costs. In the last two decades, there have been major improvements in the trauma system in the UAE, which reduced trauma mortality by nearly 60% [11, 12]. We have systematically and prospectively collected population-based trauma data in Al-Ain City over the last 20 years as part of this trauma system [13, 14]. This enables us to study the effects of these changes on the trauma of women in child-bearing age. We aimed to study the changes in the incidence, type, severity, and outcome of trauma of hospitalized child-bearing age women in Al-Ain City, UAE, over the last 15 years.

## Patients and methods

### Ethical considerations

This study was approved by the Human Research Ethics Committees of Al-Ain Health District Area (Ethical approval NO: RECA/02/44) and Al-Ain Hospital (Ethical approval NO: AAHEC-03–20-008), Al-Ain, UAE. Patients or their caregivers gave their written informed consent to use the patients' data in research.

### Data collection

Data from two separate trauma registries of Al-Ain Hospital were prospectively collected from March 2003 to March 2006, while the second period was from January 2014 to December 2017. The gap between these two periods occurred because of lack of funding. The current study is a retrospective analysis of these data. Trauma patients who were included in the registry were those who were hospitalized for more than one day and those who died on arrival at the hospital.

### Study population

This study included all women of child-bearing age (reproductive age) in the trauma registry. The child-bearing age is defined to be 15–49 years [15, 16].

### Studied variables

The variables we compared included age, nationality, method of transportation, physiological markers of injury severity including systolic blood pressure, heart rate, respiratory rate, Glasgow Coma Scale (GCS), mechanism and location of injury, anatomical markers of injury severity including new injury severity score (NISS), injury severity score (ISS), injured body regions and their Anatomical Injury Score (AIS), ICU admission, length of hospital stay, and mortality.

### Calculations

In 2006, 19.76% of the UAE population were females of child-bearing age. In comparison, 62.42% were males of the same age group (15–49 years). Only 1.2% of the population were females of 50 years old or more [17]. Al-Ain City had an estimated population of 460 000 during the first study period [18]. Accordingly, the estimated number of females aged 15–49 years in Al-Ain City during this period was 90,896 inhabitants, the estimated number of females of 50 years old or more was 5520, the estimated number of males aged 15–49 years was 287 132; while the estimated number of males of 50 years old or more was 20 654. In comparison, in 2016, Al-Ain City had a population of 766 936 inhabitants, of whom 315 182 were females (41%). Out of them 211,485 (67%) were of child-bearing age while the number of females of 50 years old or more was 21,455, the number of males aged 15–49 years was 334 984, while the number of males of 50 years old or more was 33 682 [19]. During the study periods, Al-Ain Hospital was the main trauma center in Al-Ain City, treating about 80% of the in-hospital trauma patients. Accordingly, the standardized annual incidence of hospitalized trauma child-bearing age women per 100 000 population was calculated as follows: (1.25 X annual hospital admissions)/ city population in 100 000 s.

### Statistical analysis

Continuous data were presented as mean (SD), ordinal data were presented as median (range), and categorical data were presented as number (%). Occasionally, ordinal data were also presented as mean (SD) when the comparison was statistically significant, and the median was the same in the two groups. The categorical data of the two periods were compared using Pearson's Chi-square because the number of subjects was large, with expected cell values of more than 5. Fisher's Exact test was used for comparisons when the expected value of any cell was less than 5. The continuous or ordinal data of the two periods were compared using Mann–Whitney U test. The overall significance of tables of categorical data of more than 2 × 2 was first tested. If this overall analysis was significant, then a pairwise comparison for individual categories of the two groups was done to explain the findings but not to accept overall significance. We have used Statistical Package for the Social Sciences (IBM-SPSS version 26, Chicago, Il) to perform the analysis. A p-value < 0.05 was accepted as significant.

## Results

There were 184 patients out of 2573 trauma patients in the first three-year period (7.15%) and 302 patients out of 3519 trauma patients in the second four-year period (8.58%). This gives an average of 67 patients admitted to Al-Ain Hospital annually during the first period, and an average of 75.5 patients admitted to Al-Ain Hospital annually during the second period. The calculated annual incidence of hospitalized trauma patients of child-bearing age was 84.16 per 100,000 population during the first period while it became 44.63 per 100,000 population during the second period. This resembles a 47% reduction in the incidence of trauma in this population over the last 15 years. In comparison. Furthermore, 1741 hospitalized male patients were in the age of 15–49 (67.7%) in the first period compared with 2144 (60.9%) in the second period. 237 (9.2%) male patients have an age of 50 years or more in the first period compared with 386 (11%) in the second period. There were 61 (2.37%) females of 50 years old or more in the first period compared with 182 (5.17%) in the second period. This gives an estimated incidence for the hospitalized injured male patients having an age of 15–49 years of 252.6 per 100 000 population for the first period and 200 per 100 000 population for the second period (a reduction of 20.8%). The estimated incidence for the hospitalized injured female patients having an age of 50 years old or more was 460.45 per 100 0000 population for the first period and 265.1 per 100 000 population for the second period (a reduction of 42.4%). The estimated incidence for the hospitalized injured male patients having an age of 50 years old or more was 478.11 per 100 0000 population for the first period and 358.13 per 100 000 population for the second period (a reduction of 25.1%).

**Fig. 1:**
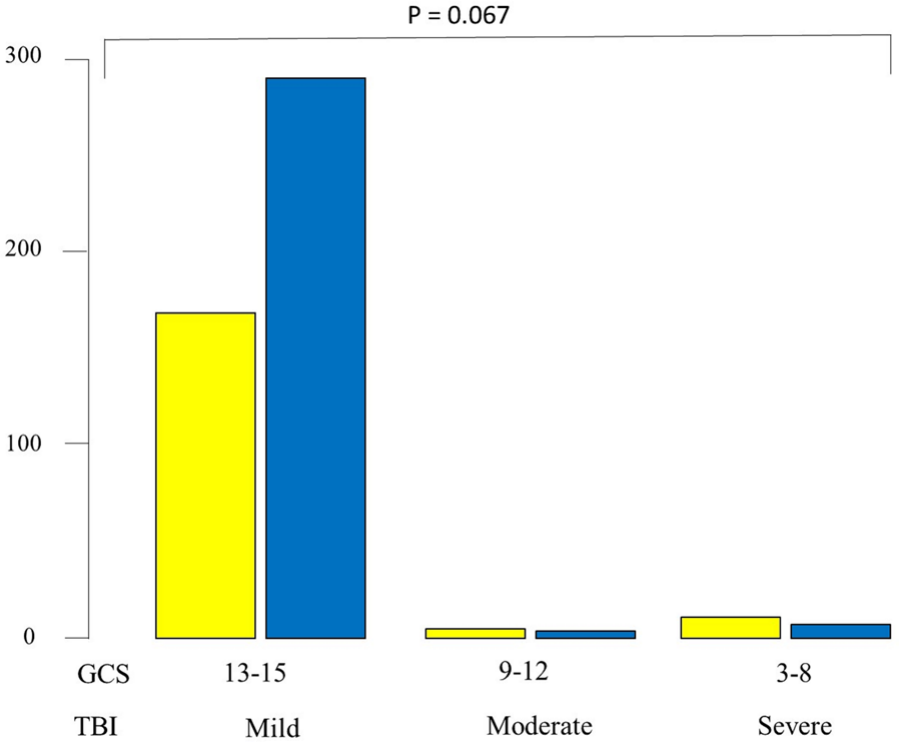
Histogram of the severity of Traumatic Brian Injury (TBI) by Glasgow Coma Score (GCS) of hospitalized child-bearing age women, Al-Ain Hospital, Al-Ain, United Arab Emirates, during the period of 2003–2006 (yellow bars, n = 184) compared with the period of 2014–2017 (blue bars, n = 302)

Table 1 shows the demography and severity markers of the patients of the two periods. There was no statistical difference in age or nationality in the two periods. Patients were transferred significantly more by ambulances to the Emergency Department during the second period (45.4% compared with 35.9% (p < 0.01). During the second period and on arrival to the Emergency Department, patients had significantly less tachycardia (mean (SD) 88.9 (15.2) compared with 95.5 (17.1) beat per minute, p < 0.001), less respiratory rate (mean (SD) 18.6 (2.4) compared with 20.2 (3.1) breath per minute, p = 0.005) and higher GCS (median (range) 15 (3–15) compared with 15 (3–5), p < 0.001. There was a strong statistical trend of mild traumatic brain injury (GCS 13–15) in the second period (p = 0.067, Fisher’s Exact test) (Fig. 1). Furthermore, those who had normal GCS of 15 were significantly higher in the second period compared with those in the first period (95.3% compared with 86.4%, p < 0.001, Fisher’s Exact test). NISS was significantly higher in the second period (median (range) NISS 5 (1–45) compared with 4 (1–75), p = 0.02). Length of hospital stay was significantly less in the second period (mean (SD) 5.6 (6.3) days compared with 10.6 (13.6) days, p < 0.0001). Mortality was similar in the two periods (1.6% compared with 1.7%, p = 0.99).

**Table 1:** Demography and severity markers of hospitalized child-bearing age women, Al-Ain Hospital, Al-Ain, United Arab Emirates, during the period of 2003–2006 (n = 184) compared with the period of 2014–2017 (n = 302)

Variable	Years 2003–2006	Years 2014–2017	P value
Age	31 (8.6)	32.3 (8.2)	0.08
UAE nationals	52 (28.3%)	90 (29.8%)	0.68
By ambulance	66 (35.9%)	137 (45.4%)	< 0.01
SBP	128.4 (20.2)	130 (18.5)	0.2
Heart rate	95.5 (17.1)	88.9 (15.2)	< 0.001
Respiratory rate	20.2 (3.1)	18.6 (2.4)	0.005
GCS	15 (3–5)	15 (3–5)	< 0.001
ISS	4 (1–41)	4 (1–41)	0.08
NISS	4 (1–75)	5 (1–45)	0.02
ICU admission	12 (6.5%)	18 (6%)	0.84
Hospital stay	10.6 (13.6)	5.6 (6.3)	< 0.001
Death	3 (1.6%)	5 (1.7%)	0.99

Data are presented as mean (SD), median (range) or number (%) as appropriate. SBP = systolic blood pressure, GCS = Glasgow coma scale, ISS = injury severity Score, NISS = new injury severity score

*p* = Pearson's Chi Square or Fisher's Exact test as appropriate for categorical data and Mann Whitney U test for ordinal or continuous data

Table 2 compares the mechanism of injury in the two periods. There were no significant differences between the two periods, with road traffic collisions as the leading cause of injury (44% and 42% respectively) followed by fall down (26.1% and 30.8% respectively). Table 3 compares the location of injuries. There was a significant difference in the location of injury (p = 0.018), with a strong trend of more home injuries in the second period (52.8% compared with 44%, p < p = 0.06). Majority of injuries occurred on streets and highways which is similar to motor vehicle collisions (44.6% and 40.2% respectively, p = 0.34).

**Table 2:** Mechanism of injury of hospitalized child-bearing age women, Al-Ain Hospital, Al-Ain, United Arab Emirates, during the period of 2003–2006 (n = 184) compared with the period of 2014–2017 (n = 302)

Mechanism	Years 2003–2006		Years 2014–2017		
	Number	%	Number	%	P value
					0.36 (overall)
MVC	81	44	125	42.1	
Fall down	48	26.1	93	30.8	
Burn	19	10.3	25	8.3	
Fall from height	16	8.7	15	5	
Machinery	3	1.6	1	0.3	
Heavy Object	2	1.1	5	1.7	
Motorcycle	2	1.1	4	1.3	
Bicycle	1	0.5	3	1	
Other	12	6.5	31	10.3	
Total	184	100	302	100	

Data are presented as number (%)

*p* = Pearson's Chi Square or Fisher's Exact test as appropriate

**Table 3:** Location of injury of hospitalized child-bearing age women, Al-Ain Hospital, Al-Ain, United Arab Emirates, during the period of 2003–2006 (n = 184) compared with the period of 2014–2017 (n = 302)

Location	Years 2003–2006		Years 2014–2017		
	Number	%	Number	%	P value
					0.018 (overall)
Street/highway	82	44.6	121	40.2	0.34
Home	81	44	159	52.8	0.06
Off road	6	3.3	4	1.3	0.19
Workplace	5	2.7	4	1.3	0.31
Public area	5	2.7	13	4.3	0.46
Other	5	2.7	0	0	0.007
Total	184	100.0	301	100.0	

Data are presented as number (%)

*p* = Pearson's Chi Square or Fisher's Exact test as appropriate

Numbers may not add to the total number of each period because of missing data

Table 4 compares injured anatomical regions and their severity between the two groups. Head injury was less common but was more severely injured in the second period (13.3% compared with 23.9%, p = 0.004; AIS 2 (1–5) compared with 1 (1–5), p = 0.025, respectively. Spine injury was more common but was less severely injured in the second period (18.9% compared with 7.1%, p < 0.001; AIS mean (SD) 1.75 (0.5) compared with 2.46 (1.1), p = 0.01. The severity of abdominal and pelvic injuries was less in the second period: AIS median (range) 1 (1–4) compared with 2 (1–3) p = 0.036. The severity of upper limb injuries was more in the second period: AIS mean (SD) 2.03 (0.67) compared with 1.7 (0.5), p = 0.005.

**Table 4:** Comparison of injured anatomical regions of hospitalized child-bearing age women, Al-Ain Hospital, Al-Ain, United Arab Emirates, during the period of 2003–2006 (n = 184) compared with the period of 2014–2017 (n = 301)

Region	Anatomical region				Abbreviated injury scale (AIS)		
	Years 2003–2006 (n = 184)	Years 2014–2017 (n = 301)	*p* value		Years 2003–2006 (n = 184)	Years 2014–2017 (n = 301)	*p* value
Head	44 (23.9%)	40 (13.3%)	0.004		1 (1–5)	2 (1–5)	0.025
Face	22 (12%)	33 (11%)	0.77		1 (1–2)	1 (1–3)	0.57
Neck	14 (7.6%)	26 (8.6%)	0.74		1 (1–1)	1 (1–1)	0.99
Chest	32 (17.4%)	48 (15.9%)	0.71		1 (1–4)	1 (1–4)	0.4
Abdomen/ pelvis	10 (5.4%)	27 (9%)	0.16		2 (1–3)	1 (1–4)	0.036
Spine*	13 (7.1%)	57 (18.9%)	< 0.001		2 (2–5) 2.46 (1.1)	2 (1–3) 1.75 (0.5)	0.01
Upper limb*	47 (25.5%)	87 (28.9%)	0.46		2 (1–3) 1.7 (0.5)	2 (1–3) 2.03 (0.67)	0.005
Lower limb	62 (33.7%)	105 (34.9%)	0.84		2 (1–4)	2 (1–3)	0.2

Data are presented as numbers (%) or median (range) as appropriate

*Data is also presented as mean (SD) when the median is the same and the comparison is statistically significant. *p* value = Fisher's Exact test

Data on pregnancy and fetal outcomes were missing in the registry. Nevertheless, there were 27 women in the second period who were referred for obstetrics and gynecology consultation. These patients had a mean (SD) age of 31.3 (7.2) years and a median (range) ISS of 1 (1–14). Seventeen were involved with RTCs, five fell on the same level, four were assaulted, and two had burns at home. Ten women had mild abdominal contusions, one had a perineal tear following a fall, and one had vaginal postcoital tear. No patient was admitted to the ICU, and none died. Only three patients were documented to be pregnant.

## Discussion

Our study has shown that the development of our trauma system during the last two decades reduced the incidence of injury in child-bearing age women by almost 50%. Prehospital care development increased the number of patients transported by ambulance and improved the vital signs when arriving at the hospital, including the systolic blood pressure, pulse, respiratory rate, and GCS. Furthermore, in-hospital care improved. Despite having more severe injuries, including the head, during the second period, in-hospital mortality remained unchanged, and the hospital stay was reduced by 50%.

During the last two decades, there have been major improvements in hospital infrastructure, standards of health care, training of trauma surgeons, promoting injury prevention, and proper legislation. Our Trauma Group, which is affiliated with the UAE University and led by academic acute care surgeons and physicians, played an important role in these developments. The Trauma Group was established in 2001 with a clear mission of improving trauma research, education, and clinical outcome. Initially, it established Al-Ain Hospital Trauma Registry in 2003 [13, 14]. This registry was very useful for trauma research that improved injury prevention strategies, mainly for road traffic collisions and work-related injuries, which reduced injury incidence [11, 12, 20]. Furthermore, the Advanced Trauma Life Support program [21] and Point-of-Care Ultrasound training courses were established and delivered both locally and internationally [22]. This was paralleled by major improvements in prehospital and in-hospital care, including damage control surgery and Point-of-Care Ultrasound [23]. The training of Emergency Medical Services has improved in the Emirate of Abu Dhabi [24]. The current study shows that the number of patients transported by ambulances to our hospital has significantly increased from 36 to 45%. Furthermore, the improved vital signs of blood pressure, heart rate, respiratory rate and GCS in the current study indicate better prehospital medical care. An important finding of clinical significance is that those who had normal GCS of 15 were significantly higher in the second period compared with those in the first period (95.3% compared with 86.4%, p < 0.001, Fisher’s Exact test) despite the significantly higher AIS of the head injury in the second period. The mechanisms of injuries and their percentages did not change over time. Road traffic collisions and falling down were on the top, causing around 70% of all injuries. Despite that, there was a very strong trend for increased home injuries (p = 0.06) which highlights the need for having a strategy to reduce the home injuries.

The pattern changes of the anatomical regions of the body overtime in this study are very interesting and challenging to interpret. In the second period, head injury was less common but more severe; spine injury was more common but less severe; abdominal and pelvic injuries were less severe, while upper limb injuries were more severe. This can be hypothetically explained by selection bias of more admitted patients who do not use seatbelts in the second period. Others who follow the traffic roles and use seatbelts will have minor injuries that do not need hospitalization. Seatbelts will anchor the chest to the seats stopping the head from hitting the windscreen in a frontal impact road traffic collisions [25, 26]. Furthermore, a seatbelt sign may be associated with severe bowel and spine injuries [26, 27]. Unfortunately, data on seatbelt usage was not available in our study to prove this.

A study from Kuwait of 728 pregnant women showed that the majority (89%) had severe injuries, with 10.7% maternal mortality and 10.7% fetal mortality. Those who wore seatbelts were only 21% of the patients, and they had minor injuries [28]. We think that this study [28] has major selection bias because majority had severe injuries. In another retrospective study from the USA of 126 pregnant women, majority used seatbelts (89%). Fetal mortality was significantly higher in those who did not use seatbelts (25% compared with 3.5%, p = 0/018) [29]. Accordingly, it is recommended that pregnant ladies should use seatbelts despite its occasional injury to the fetus [30, 31]. The seatbelts should be properly placed. The lap section should be under the bump and across the hips, while the shoulder section should pass between the two breasts and around the abdomen [32].

## Limitations of the study

It is important to stress that our study has its own limitations. *First*, our data were generated from a single hospital in Al-Ain City which has limited generalizability. *Second*, there was a time gap between the two study periods. This occurred because of lack of research funding. *Third,* both periods had missing data regarding the presence of pregnancy and its outcome. Nevertheless, we think that injury prevention in an epidemiological study should target the general child-bearing age group of women and not only those who are pregnant*. Fourth*, data on the use of seatbelts, the exact location of the passenger, and the detailed description of the collision were lacking in our registries. These data would have enabled us to explain the reason for the changes in the anatomical regions and their severity during the second period. Unfortunately, these data are usually not properly captured by trauma registries. We could previously capture these data (Type I data) only by prospective studies with specific hypothesis generating questions using focused protocols and full-time research fellows [33, 34]. Registries are at the best type II data without meticulous details which only permits retrospective analysis of prospectively collected data, similar to this study. *Finally*, our study included only hospitalized patients. It didn't include patients who did not come to the hospital, those who were treated at the Emergency Department with minor injuries, or those with severe injuries who died before arrival to the hospital. Our registries do not include the prehospital data.

## Conclusions

The incidence of trauma in hospitalized child-bearing-age women in our setting was reduced by 47% over the last 15 years. Road traffic collisions and falls were the main cause of injury. Home injuries increased over time. The mortality remained stable despite the increased severity of injured patients. More injury prevention efforts should target home injuries.

## Data Availability

No additional data is available to share with the readers. Data can be shared with the Editor of the Journal if requested.

## Abbreviations

AIS: Anatomical Injury Score
GCS: Glasgow Coma Score
ICU: Intensive Care Unit
ISS: Injury Severity Score
NISS: New Injury Severity Score
SBP: Systolic blood pressure
SD: Standard deviation
UAE: United Arab Emirates
